# Social Vulnerability and COVID-19 Vaccine in Spain

**DOI:** 10.3390/ijerph192114013

**Published:** 2022-10-27

**Authors:** Marcelino Pérez-Bermejo, Alexis Cloquell-Lozano, Carmen Moret-Tatay, Francisco Javier Arteaga-Moreno

**Affiliations:** 1 OAMI-UCV (Research Methodology Support Office), Universidad Católica de Valencia San Vicente Mártir, 46001 Valencia, Spain; 2 SONEV Research Group, School of Medicine and Health Sciences, Catholic University of Valencia, C/Quevedo nº 2, 46001 Valencia, Spain

**Keywords:** COVID-19, vaccination, social vulnerability, equity, SARS-CoV-2

## Abstract

Different analyses show that the design of vaccination policies should especially protect the most vulnerable social groups, since the level of acceptance is determined by the population’s knowledge, attitude and concerns about the safety and efficacy of vaccines. The objective of this work will be to detect the most socially vulnerable groups with respect to COVID-19 and to analyze the factors that influence predisposition to vaccination. This is a cross-sectional study using data from the Centro de Investigaciones Sociológicas (CIS) on the Effects and Consequences of Coronavirus (Study 3346 of December 2021). Sociodemographic variables (sex, age, employment status, studies and subjective class identification) were extracted, as well as the answers to the questions indicating the attitude towards vaccination, corresponding to questions 7,8,10 and 11 of the study. The most vulnerable group was lower class women (self-perceived), under 45 years of age with lower educational level, unemployed or performing unpaid work in the home. Most of them are not predisposed to vaccinate only because of the obligation to do so, mainly due to lack of belief in the power and efficacy of vaccines, as well as fear of health risks/collateral side effects. The lower vaccine uptake in this vulnerable population group may be due to a lack of awareness and lower trust in the authorities, as well as the benefits of the vaccine, which could be related to a lack of policy targeting the most socially vulnerable populations.

## 1. Introduction

Vaccination against COVID-19 has been a global challenge that has been accompanied by many fears and doubts about its efficacy, a situation that has been exacerbated by rumors and conspiracy theories [1] fueled by some public figures, which has led, in some countries, to a strong discourse against vaccination, mainly in the early discussions about vaccination against COVID-19 [2]. The need for the rapid development of vaccines has led to increased distrust [3,4] for different reasons, mainly related to possible side effects [13 of new] and distrust in the government [5], which, in some cases, has led to an increase in the rate of non-vaccination, a situation that may have been aggravated in the most vulnerable groups [6].

Different analyses show that the design of vaccination policies should especially protect the most vulnerable groups [7,8,9], increasing communication, especially of herd immunity, to protect people who cannot be vaccinated for medical reasons or children, who depend on their parents’ action [10,11,12]. All the discussed studies refer to those vulnerable for medical reasons, children or the elderly, but there are other types of social vulnerabilities that may affect concern for COVID-19 and the level of vaccine acceptance.

Vulnerability is a concept that analyzes a pre-existing condition to a new hazardous situation, such as a natural disaster, which is exacerbated by certain social factors [13,14]. This indicates that vulnerability to catastrophes such as COVID-19 is socially constructed [15,16], and shows us the social, economic, demographic and geographic characteristics that determine exposure to risk, as well as the community’s ability to deal with them. Social vulnerability, therefore, reflects the pattern of social stratification, so it is necessary to examine the social factors that explain the differential results on vaccination acceptance.

In this regard, it is clear that the level of vaccine acceptance for COVID-19 is determined by the knowledge, attitude, and concerns of the population about the safety and efficacy of vaccines [17]. Some studies have found correlations in the degrees of vaccine acceptance with respect to different sociodemographic or cultural values, such as age, purchasing power [18,19] or educational level, which is crucial for the acceptance of vaccination since the higher the level of education, the better the understanding of the messages, and the greater the ability to seek information with respect to people with lower educational level [6].

The analysis of trust and acceptance of the COVID-19 vaccine is essential for governments to understand the issues that need to be addressed to build trust. This is a complex effort that requires effective campaigns with credible scientific communication by all social groups [20,21]. Therefore, the aim of this work will be to analyze the effects of social vulnerability on predisposition to vaccination.

## 2. Materials and Methods

This is a cross-sectional study. Data from the Centro de Investigaciones Sociológicas (CIS) on the Effects and Consequences of the Coronavirus were used. The CIS is an official national organization that conducts social research through periodic surveys, whose results are published by means of barometers, which are composed of a series of fixed questions and other variables on specific topics. These barometers were designed to be statistically representative of Spanish society with a sample size of around 2500 citizens. Sampling was performed using a multi-stage stratified clustering procedure with a proportional random selection of primary (municipalities) and secondary (census districts) sampling units. The barometer coinciding with the sixth wave was chosen for the present study because it represents a change in context with the initiation of vaccines and the omicron variant. The inclusion criteria for these barometers were: a) participants had to be over 18 years of age, and b) be residents of Spain.

The barometer chosen corresponds to Study No. 3346, carried out in December 2021, when the vaccination pattern was advanced, with a sample of 2462 interviews and a margin of error of ±2.0% for a confidence level of 95.5% [22], in which the main question was the degree of affectation by the coronavirus crisis, in addition to a series of questions on the acceptance of vaccination. 

Sociodemographic variables (sex, age, employment status, studies and subjective class identification) were extracted, as well as the answers to the questions indicating attitude towards vaccination, corresponding to questions 7,8,10 and 11 of the study.

Question 7 analyzes vaccination status at the time of the survey, asking whether or not they were vaccinated. It has 4 subquestions, of which the following were used: 7a, willingness to be vaccinated; 7b the main reason for not being vaccinated. Question 8 analyzes whether respondents think that everyone should be forced to be vaccinated even if they do not want to. Question 10 analyzes whether the respondent has their own or adopted children between 5 and 11 years of age and, if so, (10a) whether he/she is willing to vaccinate them. Finally, question 11 analyzes whether, in the opinion of the respondents, vaccination of children should be mandatory.

In terms of internal consistency, Cronbach’s α was 0.777.

### Analysis

Data were entered and stored in an MS Excel file and then transferred to SPSS v.23 software (SPSS Inc., Chicago, IL, USA) for statistical analysis. Descriptive statistics (frequencies, percentages) were calculated for the sociodemographic characteristics. In addition, frequency and percentage of COVID-19 vaccine acceptance were calculated. 

Normality of the data distribution was determined using the Kolmogorov–Smirnov test. The data were presented using mean and standard deviation (SD). The data of the qualitative variables were expressed as absolute value of cases and as a percentage. The contrast between the categorical variables was performed using the Chi2 test, complemented with the analysis of standardized residuals in the case of statistically significant association. Two-sided *p* < 0.05 was considered statistically significant.

Since, in the aforementioned study no. 3346 had already reached a 70% vaccination rate with the full guideline, the variable “degree of concern” is not very discriminatory in explaining the decision in socially vulnerable groups. This is partly because we consider there to be clear differences between the variables “*what concerns you*“ and “*what affects you*” and because having a high level of concern (very much and quite a lot together) encompasses more than 80% of the sample surveyed. For this reason, we sought to replace this variable by another or others in order to explain the decision to vaccinate while identifying vulnerability profiles. For this purpose, the variable “*Emotional fears caused by the coronavirus pandemic*” was used a priori, which presents nine items (survey question no. 2). The Exploratory Factor Analysis (EFA) was used to identify the underlying factors in each vulnerable group, using principal component analysis (PCA) to extract them [23]. PCA yielded a linear combination that explained the maximum proportion of the total variance. Eigenvalues with a value >1.0 were retained in the analysis. In this analysis, a Quartimax orthogonal rotation was used to obtain the factors. To interpret the results of the factor analysis, the patterns of each factor were examined to determine the primary constituents. The two factors that were found were dichotomized to perform a bivariate analysis to identify risk factors.

## 3. Results

A total of 2462 subjects were included in the analyzed study. The mean age was 50.31 years (SD = 16.52). A total of 1227 (49.8%) were male and 1235 (50.2%) were female. Table 1 shows the analysis of sociodemographic variables.

Table 2 shows the rotated component matrix of the variable “*Emotional fears caused by the coronavirus pandemic*”. This table reflects the highest correlations of the original variables with the rotated factors, to find which ones are grouped in each of them. The extraction was performed for two components because we obtained more than 70% of the explained variance. The first factor was made up of five variables related to state of mind and the fear of becoming infected, which is why we named it “*Uncertainty about the future”*. The second factor, which is explained by the variables related to employment, personal finances and loss of family or friends, was called “*Fear of the personal and social situation”.*

Once the two factors showing the emotional fears were found, they were crossed with the sociodemographic variables to find the most vulnerable profile. Table 3 shows the analysis of the standardized residuals (only the significant ones are shown). This analysis indicates that the most vulnerable profile of this population is that of women under 45 years of age, with no or only primary education, who are unemployed and who identify themselves as being lower class or poor.

Table 4 shows an analysis of the group that was detected as the most vulnerable. Most of them were not predisposed to get vaccinated when it is their turn and will only do so because they feel obliged. The main arguments are a lack of belief in the power and effectiveness of vaccines, as well as the fear of health risks/side effects/collateral side effects.

## 4. Discussion and Conclusions

Our analysis shows a high degree of concern during the sixth wave of coronavirus, which was the one that most severely affected the Spanish population. Two main factors of concern were identified, which include fear of the future and fear of the personal or social situation of the participants. Both risk perception and distrust are considered key factors in the decision to vaccinate, together with individual freedom [17].

Several studies have shown that belonging to socially vulnerable groups greatly hinders adherence to vaccination, thereby increasing the risk of becoming infected with COVID-19 [24,25]. In addition, some characteristics, such as gender, age, income level, employment and insurance status, may result in individuals not trusting vaccines for different reasons, increasing infection rates and, as a consequence, COVID-19 mortality [26,27].

The results of our study show that educational level is key to the emotional fears caused by the pandemic, coinciding with other studies [28,29,30,31]. In addition, the most vulnerable group was lower class (self-perceived) women, with a lower educational level, unemployed or performing unpaid work at home coinciding with other studies conducted in the previous influenza H1N1 epidemic [32]. In general, women tend to be more vulnerable than men in disaster situations, partly because they are more prone to distress and threats to reproductive health, and have greater difficulties accessing economic means. Reference is also made to their lower resilience after disaster [33,34]. Women belonging to this vulnerable group do not have sufficient economic or social resources to protect themselves and have, therefore, been disproportionately affected by control measures. This suggests that they could be a target population in educational campaigns on vaccine safety and efficacy, as they are currently reluctant to be vaccinated [17].

Vulnerable groups are often undervaccinated for several reasons, including lack of awareness and uncertainty or misconceptions about the safety and efficacy of vaccination [35]. The lower vaccine uptake in the vulnerable population group analyzed in this paper may be due to such factors and their lower level of trust in the authorities [36,37]. In general, these problems may be related to the lack of policies targeting the most socially vulnerable populations. The promotion of vaccination by health personnel has been shown to be essential [38], along with adequate dissemination of clear and understandable information by public health agencies. [21,39,40,41].

## Figures and Tables

**Table 1:** Analysis of sociodemographic variables.

		Male	Female	*p*-Value *
Age (years)	18–24	85	84	0.972
	25–34	162	145	
	35–44	231	230	
	45–54	255	261	
	55–64	214	228	
	65–74	186	196	
	75–84	80	77	
	85–94	14	14	
Employment status	Working	800	748	0.000
	Retired or pensioner	306	277	
	Student	40	48	
	Unemployed	68	130	
Studies	No education	30	46	0.001
	Primary	71	109	
	Secondary 1st stage	165	209	
	Secondary 2nd stage	188	184	
	Vocational training	249	214	
	Higher	507	454	
	Others	9	13	
Subjective class identification	High/upper middle	97	66	0.006
	Middle	620	627	
	Lower middle	183	174	
	Working/Laborer	119	100	
	Low/poor	90	128	

* Chi Square test.

**Table 2:** Rotated Component Matrix of the variable “Emotional fears caused by the coronavirus pandemic”.

	Component	
	1	2
Fear of becoming ill	0.614	
Concern about measures that may limit face-to-face contact and relationships with family, friends, and neighbors	0.559	
Fear of not recovering their life as it was before the pandemic.	0.746	
Fear of no longer being able to undertake life projects such as emancipation, starting a business, or traveling.	0.622	
Concern and fear for the future	0.692	
Grief over the loss of a family member, friend or acquaintance		0.650
Concern about losing their personal job or that of a family member		0.784
Fear of the possibility of losing your personal job or that of a family member		0.767
Uneasiness about not being able to meet their expenses (mortgages, rents, loans, utilities, telephony, etc.).		0.764

**Table 3:** Analysis of the standardized residuals.

Variable	Category	Standardized Residual		*p*-Value *
		Factor 1: High uncertainty	Factor 2: High fear	
Gender	Female	3.3	2.2	<0.001
Age	Less than 45 years old	2.3	4.3	<0.001
Employment status	Unemployed or performing unpaid work at home	2.1	4.9	<0.001
Studies	No education	1.6	3.7	<0.001
	Primary	1.8	4.1	<0.001
Subjective class identification	Low/poor	2.1	4.1	<0.001

* Chi Square test.

**Table 4:** Analysis of the group detected as the most vulnerable.

Variable	Yes	No
Willingness to be vaccinated when it is your turn	12.6%	87.4%
Does not trust the vaccine	36.8%	63.2%
Do not believe it is effective	10.3%	89.7%
Fear of health risks/side effects/collateral side effects	31.0%	69%
Because they are unlikely to be contagious	4.6%	95.4
Because they have passed COVID-19	8.0%	92.0%
Prefer to wait to see how they work	5.7%	94.3%
Against all vaccines in general	3.4%	96.6%

## Data Availability

The data is available online at https://www.cis.es/cis/opencm/ES/1_encuestas/estudios/ver.jsp?estudio=14599 (accessed on 19 June 2022).
